# Incongruence between mtDNA and nuclear data in the freshwater mussel genus *Cyprogenia* (Bivalvia: Unionidae) and its impact on species delineation

**DOI:** 10.1002/ece3.2071

**Published:** 2016-03-11

**Authors:** Jer Pin Chong, John L. Harris, Kevin J. Roe

**Affiliations:** ^1^ Department of Natural Resource Ecology and ManagementIowa State UniversityAmesIowa50011; ^2^ Department of Biological SciencesArkansas State UniversityJonesboroArkansas72401

**Keywords:** Conservation, mito‐nuclear discordance, population genetics, unionid

## Abstract

Accurately identifying species is a crucial step for developing conservation strategies for freshwater mussels, one of the most imperiled faunas in North America. This study uses genetic data to re‐examine species delineation in the genus *Cyprogenia*. Historically, *Cyprogenia* found west of the Mississippi River have been ascribed to *Cyprogenia aberti* (Conrad 1850), and those east of the Mississippi River were classified as *Cyprogenia stegaria* (Rafinesque 1820). Previous studies using mitochondrial DNA sequences indicated that *C. aberti* and *C. stegaria* were not reciprocally monophyletic groups, suggesting the need for systematic revision. We generated a novel dataset consisting of 10 microsatellite loci and combined it with sequence data from the mitochondrial ND1 gene for 223 *Cyprogenia* specimens. Bayesian analysis of the ND1 nucleotide sequences identified two divergent clades that differ by 15.9%. Members of these two clades occur sympatrically across most sampling locations. In contrast, microsatellite genotypes support recognition of three allopatric clusters defined by major hydrologic basins. The divergent mitochondrial lineages are highly correlated with the color of the conglutinate lures used by mussels to attract and infest host fishes, and tests for selection at the ND1 locus were positive. We infer that the incongruence between mtDNA and microsatellite data in *Cyprogenia* may be the result of a combination of incomplete lineage sorting and balancing selection on lure color. Our results provide further evidence that mitochondrial markers are not always neutral with respect to selection, and highlight the potential problems of relying on a single‐locus‐marker for delineating species.

## Introduction

Taxonomic uncertainties are a major challenge to the conservation of endangered species because errors in the delineation of species may lead to flawed management decisions and incorrect estimates of biodiversity (Goldstein et al. 2000; Isaac et al. 2004; Frankham 2010). The delineation of species can be based on morphological, ecological, behavioral, and genetic information (Coyne and Orr 2004). Molecular taxonomy uses DNA sequences to identify molecular operational taxonomic units (MOTUs) and clarify taxonomic uncertainties by grouping morphologically cryptic organisms into distinct genetic entities (Vogler and Monaghan 2007). However, an increasing number of studies have shown that gene trees generated using mitochondrial data are often incongruent with gene trees constructed using nuclear data (e.g. Sota and Vogler 2001; Weins et al. 2010). Gene trees and species trees can be incongruent for a number of reasons including gene duplication (paralogy), introgression or hybridization between lineages (Doyle 1992; Degnan and Rosenberg 2009), and incomplete lineage sorting (Ting et al. 2008; Rodríguez et al. 2010; Hausdorf et al. 2011; Hobolth et al. 2011). Differentiating between the possible causes of incongruence is not always straightforward, and often is not attempted (Toews and Brelsford 2012).

Freshwater mussels are often considered to be keystone species in the freshwater benthic community (Aldridge et al. 2007; Geist 2010). As filter‐feeders, they serve an important functional role in the river ecosystem through enhancing nutrient cycling and increasing habitat richness for the benthic community (Vaughn and Hakenkamp 2001). Degradation of water quality and other human activities have led to the imperiled status of many freshwater species, and unionid mussels are among the most endangered faunas in the world (Williams et al. 1993; Stein and Flack 1997; Master et al. 1998; Haag 2012). Freshwater mussels are unique among bivalves in that they have a parasitic stage in their lifecycle where the larvae, termed glochidia, attach to a vertebrate host for a period of several weeks (Lefevre and Curtis 1912). Within the North American Unionidae, members have evolved many spectacular methods of attracting their fish‐hosts, including packaging their larvae to resemble food items and inducing the host to infest themselves by consuming the mock food item (Kat 1984). Prior to the advent of molecular markers, freshwater malacologists relied primarily on conchological characters (e.g. shell shape, size, and color) for mussel species identifications (Simpson 1914; Haas 1969). However, lineages identified using morphological characters alone have been shown to not always be congruent with evolutionary lineages identified using molecular markers (Roe and Lydeard 1998; Inoue et al. 2013).

**Figure 1:**
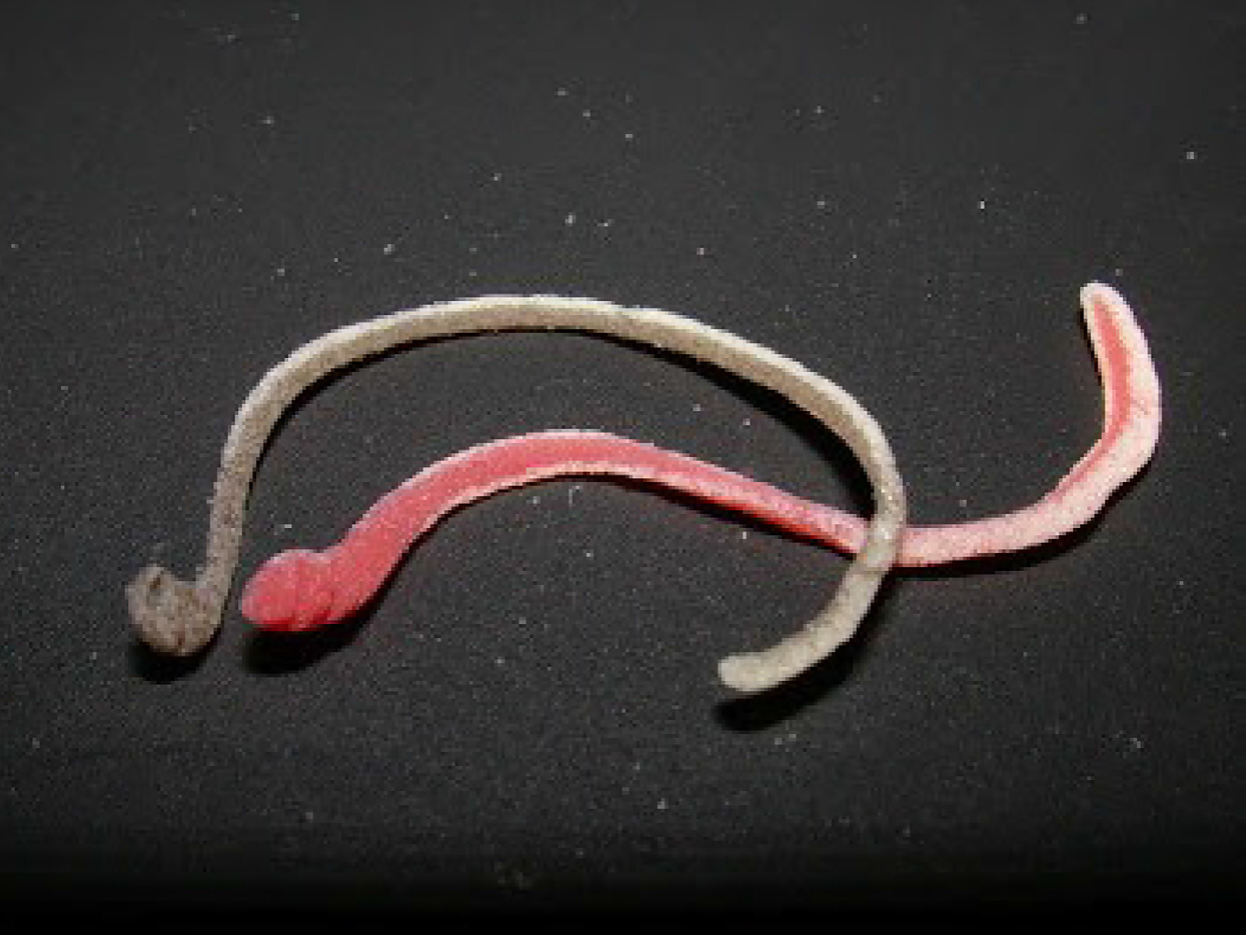
Red and brown conglutinates produced by *Cyprogenia*. Conglutinates are clusters of mussel larvae and unfertilized eggs that some mussels produced to lure host fish. Photo credit: Chris Barnhart.

The geographic range of the freshwater mussel genus *Cyprogenia* occurs within the Mississippi faunal province (Burr and Mayden 1992), and includes the Eastern, Ozark, and Ouachita highland regions that are characterized by high‐gradient streams with coarse substrates and cool water temperatures (Mayden 1988). These regions also exhibit a high degree of faunal endemism (reviewed by Hoagstrom et al. 2014). Current taxonomy recognizes two species in the genus *Cyprogenia*: the Fanshell *Cyprogenia stegaria* (Rafinesque 1820), and the Western Fanshell *Cyprogenia aberti* (Conrad 1850). *Cyprogenia stegaria* is listed as a federally endangered species (USFWS 1991) and is found east of the Mississippi River in tributaries of the Ohio River Basin, whereas the range of *C. aberti* is west of the Mississippi River in the Arkansas, White, Black, and Ouachita river basins (Oesch 1995; Harris et al. 2009). The original species descriptions indicated distinct conchological differences between these two species; however, specimens resembling intermediate forms of both species have been encountered in both the White and Ouachita river drainages in Arkansas (Harris et al. 2009). In *Cyprogenia*, mature glochidia are packaged, along with unfertilized eggs, into structures called conglutinates that resemble worms and facilitate host infection (Fig. 1). The mature glochidia are almost completely transparent, and the color of the conglutinate lure results from the pigmentation of unfertilized eggs (Eckert 2003; Barnhart et al. 2008). In *Cyprogenia*, the colors of conglutinates observed to date include brown, red, and white.

Previous molecular studies of *Cyprogenia* using mitochondrial DNA (mtDNA) sequences have indicated that both *C. aberti* and *C. stegaria* are not reciprocally monophyletic groups (Serb 2006; Grobler et al. 2011). Serb (2006) reported two monophyletic groups within *Cyprogenia,* but each clade included individuals of both nominal species. The two evolutionarily distinct clades (14% sequence divergence) reported were sympatric in several drainages. In addition, these two mtDNA lineages seemed to be correlated with the color of the conglutinate lures. *C. aberti* specimens that produced red‐colored conglutinates grouped into one mtDNA clade, and those with brown conglutinates grouped into the other clade (Serb and Barnhart 2008). These observations led to the development of the hypothesis that the mitochondrial lineages of *Cyprogenia* might be maintained by negative frequency‐dependent selection by host fish (Barnhart et al. 2008; Serb and Barnhart 2008). Under this hypothesis host fish learn to avoid conglutinates of the abundant color, and instead select the less common form. Grobler et al. (2011) obtained similar results for their mtDNA analyses, but the microsatellite markers they included (only for the *C. stegaria* specimens) showed little differentiation.

Previous studies have raised doubts about the validity of the two species of *Cyprogenia*. For this study, we employed both mtDNA sequences and nuclear microsatellite loci in an explicit test of alternative hypotheses concerning the number of evolutionary entities within the genus. Our study improves on previous efforts in that we have combined both mitochondrial and nuclear data for both *C. aberti* and *C. stegaria* samples, which had not been achieved previously, and increased the numbers of sampling sites and sample sizes over previous efforts. Finally, we discuss the implications of our findings regarding the reliability of mtDNA markers and for the conservation and management of *Cyprogenia*.

## Materials and Methods

### Sample collection and DNA extraction

**Figure 2:**
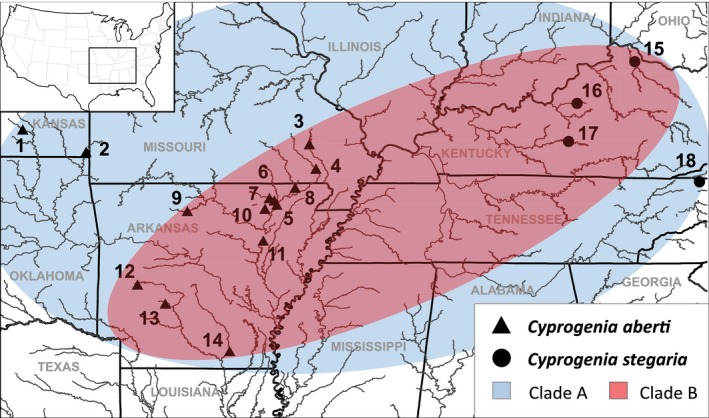
Map of *Cyprogenia* mtDNA lineage distribution in our sampling locations. *Cyprogenia aberti* (triangles) were collected from Arkansas, Missouri, and Kansas. *Cyprogenia stegaria* (circles) were collected from Kentucky and Tennessee. Drainages of sampling sites are labeled: (1) Fall, (2) Spring River, Kansas, (3) St. Francis, (4,5) Black, (6,7) Spring River, Arkansas, (8) Current, (9) Buffalo, (10) Strawberry, (11) White, (12) Ouachita, (13) Caddo, (14) Saline, (15) Licking, (16) Salt, (17) Green, (18) Clinch. *Cyprogenia* mtDNA Clade A (blue) occurred in all sampling sites. Clade B (red) co‐occurred with Clade A in most sampling sites except in Kansas and Tennessee populations.

A total of 223 *Cyprogenia* samples were included in this study. One hundred and forty‐four *Cyprogenia aberti* individuals were collected in 2010 and 2011 in collaboration with the Arkansas Game and Fish Commission, Arkansas State Highway and Transportation Department, Missouri Department of Conservation, and U.S. Fish and Wildlife Service (Fig. 2 and Table S1, Supporting Information). Samples for DNA extraction were collected nondestructively using cytology brushes (Henley et al. 2006), and genomic DNA was extracted using the Puregene Buccal Cell Kit (Qiagen, Hilden, Germany). The color of the conglutinate lures was recorded if female individuals were collected during the breeding season. Additional genomic DNA was obtained from 26 individuals (24 *C. aberti* and two *C. stegaria*), from Serb (2006), and 53 *C. stegaria* individuals from Grobler et al. (2011) (Table S1, Supporting Information).

### Mitochondrial DNA sequencing and analysis

A ~900 base‐pair fragment of the first subunit of the mitochondrial NADH dehydrogenase (ND1) gene was successfully amplified via PCR for 206 *Cyprogenia* (157 *C. aberti* and 49 *C. stegaria*) using primers described in Serb (2006). PCR was conducted using a 25 *μ*L reaction volume, with 0.2 mmol/L dNTPs, 1x Biolase buffer, 2.5 mmol/L MgCl_2_, 0.8 *μ*mol/L primers, and 1.25 U Biolase *Taq* polymerase (Bioline Inc., Luckenwalde, Germany). Cycling parameters included an initial denaturation at 95°C for 5 min, followed by 35 cycles of 94°C for 30 sec, 52°C for 30 sec, 72°C for 60 sec, and a final extension of 72°C for 4 min. PCR products were purified using ExoSAP‐IT reagent (USB Corp., Santa Clara, CA, USA) and were bi‐directionally sequenced on an ABI 3730xl DNA Analyzer at the Iowa State University (ISU) DNA Facility. Thirty‐eight *C. stegaria* ND1 haplotype sequences from Grobler et al. (2011) were downloaded from GenBank to obtain a total of 244 *Cyprogenia* sequences. In addition, ND1 sequences for 18 outgroup taxa were also obtained from GenBank and included in the phylogenetic analysis (Table S2, Supporting Information).

ND1 sequences were aligned using ClustalW and default parameters as implemented in the software Geneious Pro v.5.5.6 (Drummond et al. 2010). Sequences were translated into amino acids in order to check for stop codons, indels, and ambiguous nucleotides. MrModeltest v.2.3 (Nylander 2004) was used to perform hierarchical likelihood ratio tests to determine the appropriate nucleotide substitution models for Bayesian analysis. Bayesian inference was conducted in MrBayes v.3.2.1 (Ronquist and Huelsenbeck 2003) using Markov chain Monte Carlo simulations of 22 million generations with tree sampling every 100 generations and a burn‐in of 100,000 generations. The burn‐in and length of MCMC simulations was determined by the stability of the mean standard deviation of split frequencies between two independent runs. Each run consisted of four chains (three hot and one cold) with a temperature difference of 0.2. A consensus tree was constructed by including all the post burn‐in sampled trees, with nodal support indicated by posterior probabilities. Pairwise genetic distances were calculated within and between each clade to evaluate sequence dissimilarity using the Kimura 2‐parameter model in program MEGA v.5.10 (Tamura et al. 2011).

Phylogenetic constraint analyses were performed to test whether two alternate tree topologies were as good or better fit to the data than the optimal trees obtained from the Bayesian analysis. The first alternative tree was constrained based on current taxonomy, by forcing all individuals east of the Mississippi River and all individuals west of the Mississippi River to form separate monophyletic groups. The second constraint tree forced individuals from the same hydrologic basins to form monophyletic groups. Constrained trees were generated in MrBayes v.3.2.1 as above, but for 10 million generations and burn‐in of 50,000. The Shimodaira‐Hasegawa test (Shimodaira and Hasegawa 1999) test implemented in PAUP* v. 4.0b10 (Swofford 2002) was used to compare the likelihoods of these two constraint trees with the unconstrained tree that we had generated earlier with Bayesian inference to see which topology was better supported.

DNA sequences were converted into haplotypes using program DnaSP v.5.10.01 (Librado and Rozas 2009). Nucleotide diversity (*π*) and haplotype diversity (*H*_d_) were estimated for each population. A haplotype network employing the optimality criteria of parsimony was generated in the program Network v.4.613 and Network Publisher v.2.0.0.1 (Fluxus Technology, Ltd., State College, PA). For samples for which we were able to collect both mtDNA data and conglutinate color information, a Yule's *Q* contingency coefficient was calculated to determine the correlation between the mussel conglutinate colors and membership within the two mtDNA clades (Yule 1900). Neutrality of the ND1 sequences was examined to see if the gene is under selection as has been previously hypothesized. Deviations from neutrality were examined using the codon based *Z*‐test of selection [*H*_0:_ *d*_N_ = *d*_S_] (Nei and Gojobori 1986) and Tajima's *D* (Tajima 1989) implemented in MEGA v.5.10 (Tamura et al. 2011) and DnaSP v.5 (Librado and Rozas 2009) respectively.

### Microsatellite genotyping and analysis

Ten microsatellite loci (Ecap1, Ecap2, Ecap4, Ecap5, Ecap6, Ecap7, Ecap8, Ecap10, PfaD06, LabD213) (Eackles and King 2002; Galbraith et al. 2011) were amplified for 216 individuals from 12 populations. An M13‐tag (5′‐AGGGTTTTCCCAGTCACGACGTT‐3′) was added to the 5′ end of the forward primer for all loci. For some microsatellite loci, an additional sequence (GTTTCTT) was added to the 5′ end of the reverse primer to promote adenylation and reduce one base pair stutter (Brownstein et al. 1996). Microsatellite reactions consisted of 0.2 mmol/L dNTPs, 1x Biolase buffer, 1.5 mmol/L MgCl_2_, 0.2 *μ*mol/L of M13 dye‐labeled primer and nontagged reverse primer, 0.02 *μ*mol/L of M13‐tagged forward primer, 0.25 U Biolase *Taq* polymerase, and 15 ng template DNA in a 20 μL total reaction volume. PCR reactions were performed with initial denaturation at 95°C for 5 min, followed by 35 cycles of 94°C for 30 sec, 55°C annealing temperature for 30 sec (except for marker Ecap8, where we used 60°C), 72°C for 30 sec, and a final extension of 72°C for 4 min. Ten percent of the samples were chosen randomly and replicated for each microsatellite locus to characterize and reduce genotyping errors as described in Meirmans (2015). PCR products for microsatellite genotyping were sent to the ISU DNA Facility and analyzed using an ABI 3730 DNA Analyzer.

Microsatellite alleles were scored using GeneMarker^™^ Software (Softgenetics, State College, PA). Micro‐Checker (Van Oosterhout et al. 2004) was used to examine the data from each marker for genotyping errors and presence of null alleles. Allelic richness, heterozygosity, and inbreeding coefficient were estimated for samples collected from the same locality using GenAlEx v.6.5 (Peakall and Smouse 2006). Sampling sites with fewer than five individuals were not included in these analyses because allele frequencies could not be properly estimated due to low sample size. Deviation from Hardy**–**Weinberg equilibrium and linkage disequilibrium were tested for all loci in GENEPOP v.3.3 (Raymond and Rousset 1995). BOTTLENECK v.1.2 (Piry et al. 1999) was used to detect rapid changes in population size due to demographic factors. The two‐phase model (TPM) with a fixed proportion of 70% single‐step model (SSM) and 30% variance of geometric distribution was implemented with 1000 iterations for sampling sites with more than eight individuals. We used STRUCTURE v.2.2 (Pritchard et al. 2000) to assign individuals into populations. A total of 100,000 MCMC replicates were performed using an admixture model with burn‐in of 50,000 runs. The correlated allele frequencies model was selected to detect refined population structure. The most likely number of populations was estimated by determining the likelihood of K following Evanno et al. (2005) using STRUCTURE HARVESTER (Earl and vonHoldt 2012); the number of *K* tested ranged from *K* = 1 to *K* = 20. CLUMPP v.1.1.2 (Jakobsson and Rosenberg 2007) and DISTRUCT v.1.1 (Rosenberg 2004) were used to construct the STRUCTURE barplot to infer the genetic structure of both species. We performed AMOVA of the genotype data in GenAlEx to determine whether partitioning of the genetic variation at the 10 microsatellite loci was consistent with the pattern obtained in the optimal mtDNA tree or either constrained tree (individuals constrained based on taxonomy, clades or hydrologic basins). The Akaike information criterion (AICc) was used to choose the best‐supported hypothesis, following Halverson et al. (2008), which would select the model with the regional factor that contributed most to the total amount of genetic differentiation. Pairwise genetic differentiation between groups was estimated using *F*‐statistics, standardized analogs (*F’*_ST_) and Jost's *D* (Weir and Cockerham 1984; Jost 2008; Bird et al. 2011; Meirmans and Hedrick 2011) using the program GenoDive v.2.0b23 (Meirmans and Van Tienderen 2004).

## Results

### Mitochondrial DNA analyses

**Figure 3:**
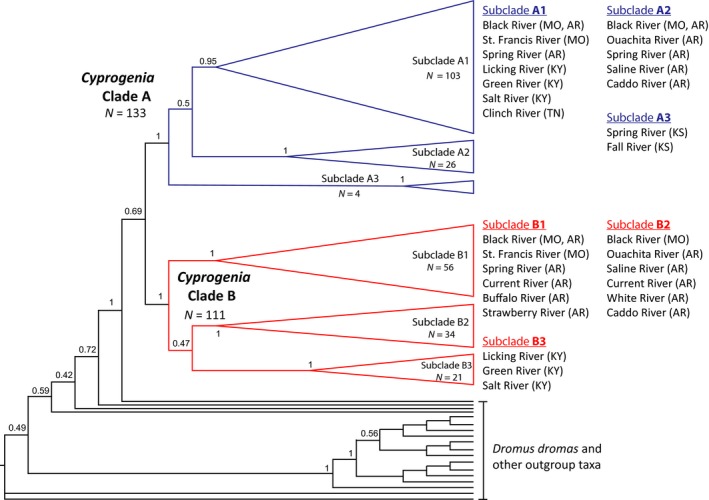
Bayesian inference gene tree based on the mitochondrial ND1 gene of two *Cyprogenia* species (−ln *L* = 6927.24). Two major mtDNA clades (A and B) differed by a genetic distance of 15.9%. Clade A includes 133 sequences and Clade B includes 111 sequences. Three subclades were identified in both Clade A and Clade B, with genetic distances ranged from 2~4% among subclades. Geographical distributions of samples for each subclade were listed by river drainage (State). Colors of clades are the same as in Figure 2.

The GenBank accession numbers for all new sequences including samples resequenced from Serb (2006) were KU687119–KU687320. Our phylogenetic analysis of the ND1 data resulted in a monophyletic *Cyprogenia* that consisted of two deeply diverged clades that were distributed sympatrically across most of our sampling sites (Fig. 2). These two clades differed by an average genetic distance of 15.9%, and are referred to as Clade A and Clade B following Serb (2006) (Fig. 3). Clades A and B consisted of 133 and 111 individual ND1 sequences respectively. Both Clade A and Clade B included individuals that were morphologically identified as *C. aberti* and *C. stegaria*. Clade A is distributed across all our sampling locations (Fig. 2). Clade B co‐occurs with Clade A at all sites, with the exception of two sites in Kansas and one in Tennessee. Three subclades within each major clade were identified using the posterior probabilities from the Bayesian inference and genetic distances (Fig. 3). These subclades were estimated to be 2–4% divergent. The first subclade within Clade A (Subclade A1) was the most widely distributed, with individuals in seven drainages: Black, St. Francis, Spring (AR), Licking, Green, Salt, and Clinch rivers. The second subclade (Subclade A2) included populations from the Black, Ouachita, Spring (AR), Saline, and Caddo rivers. Members in the third subclade (Subclade A3) were found only in the Fall and Spring (KS) rivers in Kansas. Clade B also consisted of three subclades (Fig. 3). Subclade B1 included samples from the Black, St. Francis, Spring (AR), Current, Buffalo, and Strawberry rivers. Subclade B2 included individuals from the Black, Ouachita, Saline, Current, White, and Caddo rivers. Subclade B3 was limited to Kentucky populations from the Licking, Green, and Salt rivers.

**Figure 4:**
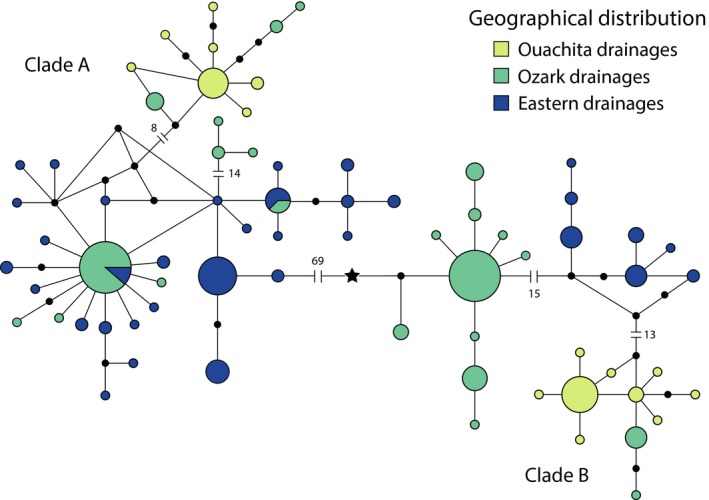
Median‐joining haplotype network for the ND1 sequences. Haplotypes are color‐coded based on major hydrologic basins. Ouachita drainages (yellow), Ozark drainages (green), and Eastern drainages (blue) includes the Licking, Green, Salt, and Clinch rivers. Each node represents a unique haplotype, and node size indicates the number of individuals sharing the same haplotype. Black nodes represent inferred mutational events occurring between haplotypes. Star symbol indicates the separation point between mtNDA clades A and B. For *n* > 4 mutational events, numbers next to interrupted lines are used to indicate the number of mutations.

All mtDNA sequences included in this study were grouped into 71 haplotypes. Overall nucleotide diversity (*π*) was 0.072 with high haplotype diversity *H*_d_ = 0.94. The numbers of haplotypes, nucleotide and haplotype diversity are presented in Table 1. Despite the co‐occurrence of individuals from Clades A and B across the range of *Cyprogenia*, most of the haplotypes were observed in a single drainage basin, with only two of 71 haplotypes shared between the Ozark highlands (Black, St. Francis, and Spring AR rivers) and Eastern highlands (Licking, Green, Salt, and Clinch rivers) (Fig. 4). Results of the Shimodaira and Hasegawa (1999) test comparing the optimal mtDNA tree recovered from the phylogenetic analysis with the trees constrained by either basins or taxonomy indicated that the unconstrained tree was significantly better than either constrained topology (*P *<* *0.001) (Table 2).

**Table 1:** Summary of mtDNA ND1 gene and microsatellite diversity from *Cyprogenia* samples grouped according to current taxonomy or corresponding to the clusters resulting from the STRUCTURE analysis. Site corresponds to ID in Fig. 2 and Table S1 in Supporting Information. Sites with fewer than five individuals were not included in this table. mtDNA sequences of 194 samples were grouped into 71 haplotypes. The number of individuals (*N*_seq_), number of haplotypes (*H*), nucleotide diversity (*π*) and haplotype diversity (*H*_d_) are listed below. Microsatellite genotyping data presented here were collected from 208 individuals. The number of individuals (*N*_msat_), allele richness (*A*), observed heterozygosity (*H*_O_), Nei's (1978) unbiased expected heterozygosity (*H*_E_), and inbreeding coefficient (*F*_IS_). The number of individuals from each sampling locality assigned to population clusters by STRUCTURE are listed in parentheses

Current taxonomy/clusters	Site	Region/Basin	mtDNA ND1 sequencing				Microsatellite genotyping					
			*N*_seq_	*H*	*π*	*H*_d_	*N*_msat_	*A*	*H*_O_	*H*_E_	*F*_IS_	STRUCTURE cluster
*C. aberti*	3	Ozark	28	6	0.0315	0.659	31	12	0.694	0.738	0.064	Ozark (30), Eastern (1)
	4	Ozark	28	9	0.0642	0.786	29	14	0.806	0.810	0.000	Ozark
	5	Ozark	19	8	0.0561	0.719	22	13	0.757	0.777	0.014	Ozark
	6	Ozark	8	6	0.0341	0.893	8	8	0.700	0.849	0.137	Ozark (7), Ouachita (1)
	7	Ozark	17	10	0.0626	0.904	18	12	0.789	0.811	0.000	Ozark
	12	Ouachita	27	13	0.0563	0.838	31	12	0.700	0.717	0.021	Ouachita (29), Ozark (1), Eastern (1)
	13	Ouachita	5	4	0.0758	0.900	5	5	0.655	0.701	0.000	Ouachita
	14	Ouachita	13	7	0.0707	0.872	13	9	0.623	0.677	0.061	Ouachita
*C. stegaria*	15	Eastern	23	8	0.0571	0.830	21	12	0.743	0.773	0.014	Eastern
	16	Eastern	8	5	0.0685	0.893	10	9	0.750	0.792	0.000	Eastern
	17	Eastern	8	6	0.0549	0.929	10	9	0.770	0.794	0.000	Eastern
	18	Eastern	10	8	0.0046	0.956	10	10	0.802	0.852	0.002	Eastern
Ozark cluster	3–7	Ozark	100	27	0.065	0.865	108	20	0.753	0.794	0.057	Ozark (106), Ouachita (1), Eastern (1)
Ouachita cluster	12–14	Ouachita	45	21	0.062	0.908	49	13	0.675	0.723	0.061	Ouachita (47), Ozark (1), Eastern (1)
Eastern cluster	15–18	Eastern	49	24	0.055	0.940	51	17	0.760	0.805	0.049	Eastern (51)

**Table 2:** The results of Shimodaira‐Hasegawa Test (SH‐test) on the likelihoods of constraint and unconstraint trees corresponding to three a *priori* models. The first model constrained individuals into *C. aberti* and *C. stegaria* based on current taxonomy identified by morphological and geographical locations. The second model was an unconstrained tree that identified two distinct mtDNA clades using optimal Bayesian analysis. The third model constrained individuals based on major hydrologic basins

Model	Tree constraint	−ln *L*	Diff in ln *L*	*P*
1	Taxa (east, west)	7224	443	<0.001
2	mtDNA clades A and B (unconstrained)	6781	(best supported)	
3	Hydrologic basins	7715	934	<0.001

Conglutinate color information was recorded for gravid female mussels during sampling. Due to the time of year when samples were collected, gravid female mussels were observed only when sampling the populations from the Ozark and Ouachita regions. Of the 180 samples that were collected, conglutinate color was observed for 53 individuals (29.4%). Of these, 21 individuals (39.6%) had red conglutinates, and 32 (60.4%) had brown conglutinates. No white conglutinates were observed during our sampling. 20 of the 21 females with red conglutinates were placed in clade A, and 31 of the 32 females with brown conglutinates were placed in clade B. Based on the Yule's *Q* contingency coefficient, mussel conglutinate colors and the mtDNA lineages (A vs. B) were strongly correlated (*Q *=* *0.997). Analysis of the ND1 sequences for evidence of deviation from neutrality using Tajima's *D* statistic (*D *=* *3.10, *P *<* *0.001) and the codon based Z‐test (*Z* = −11.22, *P *<* *0.001) both rejected the null hypothesis of neutrality.

### Microsatellite genotyping analyses

**Figure 5:**
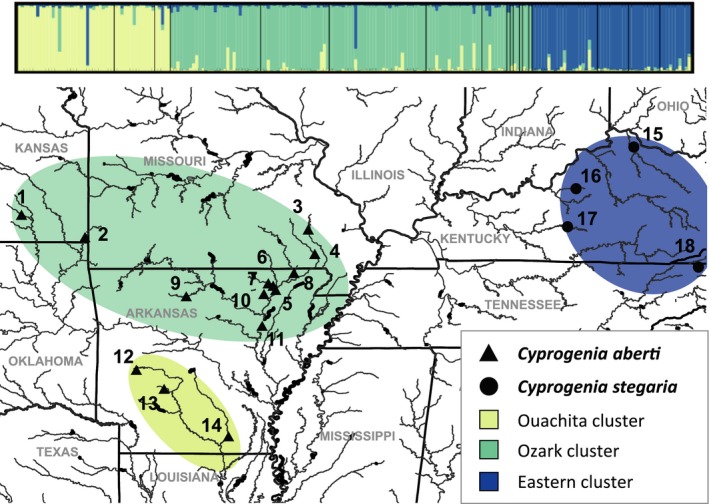
Geographic distribution of *Cyprogenia* ESUs based on results of STRUCTURE analysis. Bar plot generated from the microsatellite data shows *Cyprogenia* individuals grouped into three clusters (Ouachita, Ozark, and Eastern) corresponding to hydrologic basins.

The results from examination of the microsatellite data using Micro‐Checker (Van Oosterhout et al. 2004) indicated that locus Ecap1 may include a null allele. In order to assess the potential impact of including this locus in future analyses, we performed STRUCTURE and AMOVA analyses with and without including the Ecap1 locus. Inclusion of the Ecap1 locus did not alter the results of either analysis, and so this locus was retained in all further analyses. All loci were tested for Hardy**–**Weinberg equilibrium (HWE) using exact test and the default settings of GENEPOP (Guo and Thompson 1992). Hardy**–**Weinberg disequilibrium was detected at sites 3, 4, for locus LabD213, and at site12 for locus Ecap1 after applying the Bonferroni correction (*P *<* *0.0004). Gametic disequilibrium was not evident in any pairs of loci (*P *>* *0.05), and no evidence of a bottleneck was found in any population. Standard population genetic measures including allelic richness, genetic diversity, and inbreeding coefficient were estimated for each site (Table 1). The STRUCTURE analysis identified three clusters (*K* = 3) that corresponded to the hydrologic basins (Fig. 5). Only four of 208 individuals included in the study did not group according to their geographical region (Table 1). The first cluster (Ouachita) consisted of 49 individuals mainly from the Ouachita and Saline rivers. The second cluster (Ozark) was the largest, with 108 samples from the Black, St. Francis, and Spring (AR) rivers. The third cluster (Eastern) included 51 samples from four rivers: the Licking, Green, Salt, and Clinch rivers. Following Halverson et al. (2008), we compared the results from the AMOVA analysis to alternative hypotheses by grouping samples to reflect the same hypotheses tested for the mtDNA data (taxa, clades, or basins) to determine which contributed the most to the total amount of nuclear genetic differentiation. The Akaike information criterion (AICc) clearly indicated that grouping samples based on hydrologic basins was significantly better than the other models (Table 3).

**Table 3:** Results of AMOVA analysis performed on individuals grouped according to three a *priori* models. The Akaike information criterion (AICc) indicated that hydrologic basin was the regional factor that contributed the most to the total amount of genetic differentiation in microsatellite data. The Akaike information criterion (AICc) indicated that hydrologic basin (model assigned with *) was the regional factor that contributed the most to the total amount of genetic differentiation in microsatellite data

Model	Region	*n*	SSR	Est. variance	% variance	*F’*_RT_	*P*	AICc
1	Taxa (east, west)	208	877.6	0.392	9	0.484	0.001	307.65
2	mtDNA clades	192	910.4	0	0	0	0.999	307.04
3	Hydrologic basins	208	819.6	0.452	10	0.480	0.001	293.43*

Estimates of population differentiation indicated that the Ouachita cluster was genetically more similar to the Ozark cluster than the geographically more distant Eastern cluster. Pairwise *F*_ST_ among the three distinct groups of mussels identified by the microsatellite data were estimated to be 0.09 to 0.15 among clusters (Table 4). However, *F*_ST_ has been shown to provide underestimates of genetic differentiation when using highly polymorphic loci such as microsatellites (Jost 2008; Meirmans and Hedrick 2011). Unbiased estimators such as *F’*_ST_ and Jost's *D* have been shown to provide more accurate measures of genetic differentiation when polymorphism is high. The values for *F’*_ST_ and Jost's *D* (Table 4) indicated that the pairwise genetic differentiation among clusters was dramatically higher (0.4–0.6), suggesting very limited recent gene flow between different hydrologic basins.

**Table 4:** Genetic differentiation estimators *F*_ST_, *F’*_ST_ and Jost's *D* calculated for *Cyprogenia* populations among Ouachita, Ozark, and Eastern (Licking, Green, Salt, and Clinch rivers) clusters

Regional/Basin	*N*	*F*_ST_ (Wright 1951)			*F’*_ST_ (Meirmans 2006)			Jost's *D* (Jost 2008)		
		Ouachita	Ozark	Eastern	Ouachita	Ozark	Eastern	Ouachita	Ozark	Eastern
Ouachita	49	0.00	0.09	0.14	0.00	0.40	0.60	0.00	0.33	0.53
Ozark	108	0.09	0.00	0.11	0.40	0.00	0.55	0.33	0.00	0.49
Eastern	51	0.14	0.11	0.00	0.60	0.55	0.00	0.53	0.49	0.00

## Discussion

### Discordance between mtDNA and nuclear markers

Mitochondrial gene sequences have been a standard molecular marker for inferring phylogenetic relationships between species and phylogeographic patterns within species. However, in recent years, an increasing number of studies have documented incongruence between patterns produced by analysis of mtDNA and nuclear DNA (Lu et al. 2001; Sota and Vogler 2001; McCracken and Sorenson 2005; Rodríguez et al. 2010; Toews et al. 2014). In order to investigate the apparent incongruence further we conducted additional analyses on each data set. We tested the congruency of the results of the mtDNA data by using constraint analysis to impose the results of the microsatellite analysis onto the mtDNA data set, and we tested the microsatellite data by grouping the genotypes according to the results of the mtDNA analysis, conducting another AMOVA analysis, and comparing these results to the original analysis using the AICc. The results of the Shimodaira‐Hasegawa test of the DNA sequences, and the AICc test for the microsatellite data clearly showed that the patterns recovered from the analysis of the mtDNA data and the microsatellite data were incongruent with each other. In a recent review of this topic, Toews and Brelsford (2012) identified 126 published cases of discordance between phylogeographic patterns produced by mtDNA and nuclear DNA markers. In the cases they reviewed, mito‐nuclear discordance was reported by researchers to be due to several different factors, including incomplete lineage sorting, introgressive hybridization, and retention of ancestral polymorphisms (Ting et al. 2008; Rodríguez et al. 2010; Hausdorf et al. 2011). An additional potential source of incongruence unique to some bivalve lineages is doubly uniparental inheritance (DUI) of mitochondria, in which sex‐associated mitochondrial lineages are inherited maternally or paternally (Zouros et al. 1994; Liu et al. 1996). In DUI, male mussels contain both male and female mtDNA lineages, although the male mtDNA lineage is largely restricted to the gonads, and the female mtDNA lineage is found in the somatic tissue. Female mussels possess only the female mtDNA lineage in both gonads and somatic tissue. Incongruence between our mtDNA and microsatellites results could be obtained if the male mtDNA lineage was accidently amplified and sequenced for a subset of samples. The resulting mtDNA phylogeny would then consist of two divergent lineages (one male and the other female). We are confident that this is not the case in our study. We obtained samples for DNA extraction by collecting cells from the mantle and foot using cytology brushes, thus avoiding gonadal tissue. Additionally, male and female mtDNA lineages in unionid mussels have been shown to evolve at dramatically different rates, and differences between the two lineages can exceed 30% sequence divergence (Breton et al. 2007). A phylogenetic comparison of male and female mtDNA sequences in *Cyprogenia* (not shown) indicates all mtDNA sequences included in this study are from the female lineage.

Biased introgression in mtDNA can also result in the mito‐nuclear discordance, and could be caused by sex‐biased dispersal, assortative mating, and sexual selection (Chan and Levin 2005). Based on the biology of freshwater mussels, sex‐biased dispersal is an unlikely explanation for the observed incongruence between the two markers. Like most freshwater mussel species, *Cyprogenia* are dioecious (Haag 2012), but there is no evidence that male and female glochidia larvae are dispersed different distances by their host fishes. Sex‐biased dispersal has also not been documented in adult mussels. Similarly, male freshwater mussels broadcast sperm, and no mechanisms whereby female mussels can “choose” between the sperm of different males have been proposed, which appears to eliminate sexual selection and assortative mating as explanations for the observed incongruence.

In the process of incomplete lineage sorting, the discordance between the patterns recovered for mtDNA and nuclear DNA may be explained by the different rates at which the two types of markers fix new mutations (Martinsen et al. 2001; Ballard and Whitlock 2004). Because of its smaller effective population size, mtDNA should fix new mutations and undergo lineage sorting faster than nuclear DNA (Ballard and Whitlock 2004). In *Cyprogenia,* however, it appears that it is in fact the mtDNA that is exhibiting incomplete lineage sorting. One way to distinguish discordance arising from incomplete lineage sorting from discordance arising from other factors is that incomplete lineage sorting should not produce predictable biogeographic patterns (Funk and Omland 2003; Toews and Brelsford 2012). Our microsatellite data strongly indicated that there are three distinct nuclear DNA clusters within *Cyprogenia* that conform to biogeographic provinces (Ozark, Ouachita, and Eastern basins), with two divergent mtDNA lineages occurring within each of these clusters (Tables 3 and 4). In contrast, the 71 mtDNA haplotypes did not display any strong biogeographic patterns (Fig. 4); therefore, we cannot rule out the possibility that the mito‐nuclear discordance in *Cyprogenia* was due to incomplete lineage sorting of mtDNA.

An increasing number of studies have indicated that mtDNA appears to be under selection (e.g., Grant et al. 2006; Stewart et al. 2008), and it is conceivable that the disparate mtDNA lineages in *Cyprogenia* have been maintained in sympatry via selection. It was suggested previously (Barnhart et al. 2008; Serb and Barnhart 2008) that the mtDNA lineages in *Cyprogenia* were somehow linked to the colors of the conglutinate lures, and the color polymorphism present in populations may maintained by negative frequency‐dependent selection on conglutinates by the host fishes. The results of our more extensive examination of conglutinate color with a larger sample size concurred with Serb and Barnhart (2008) that mtDNA clade membership was highly correlated with the color of the conglutinates. Furthermore, additional evidence from the codon based Z test and the Tajima's test indicates that the two mtDNA clades are under selection. Positive Tajima's *D* values are consistent with balancing (negative frequency dependent) selection, but can also result from demographic effects, such as a recent bottleneck, or population subdivision (Maruyama and Fuerst 1985; Simonsen et al. 1995). Demographic causes should leave their signature on the nuclear genome as well as the mitochondrial genome. Our tests of the nuclear microsatellite data for a recent bottleneck, however, did not support a demographic explanation for the divergent haplotypes: tests for a bottleneck were negative, and population subdivision was minimal when specimens were grouped according to conglutinate color (*F*_ST_ = 0.005). Taken together, these results are consistent with the hypothesis that the divergent mtDNA lineages are being maintained in sympatry by negative frequency dependent selection imposed by host fish on the conglutinate color in *Cyprogenia*.

Our microsatellite data support recognizing three independent evolutionary lineages in genus *Cyprogenia* that correspond to the Ozark, Ouachita, and Eastern Highland regions of North America respectively (Fig. 5). This same biogeographic pattern has been observed in a number of other aquatic taxa that are also highland endemics (e.g., Strange and Burr 1997; Ray et al. 2006; Berendzen et al. 2008). The Central Highlands of North America once consisted of a single region characterized by clear, cool high‐gradient streams that subsequently was fragmented by a series of glacial cycles (Thornbury 1965; Pflieger 1971). The highlands became isolated into three major areas separated by intervening lowlands, and today are composed of the Ozark and Ouachita highlands west of the Mississippi River, and the Eastern Highlands containing the Appalachian Mountains east of the Mississippi River (Mayden 1988). Gene flow of freshwater mussels is considered to depend largely on the dispersal ability of their host fishes (Watters 1992; Haag and Warren 1997). Host‐fish dispersal in riverine ecosystems often can be limited by physiographic barriers such as natural features (falls, etc.) or unsuitable habitat. These barriers may create biogeographic islands by reducing gene flow among hydrologic basins, resulting in population structure that reflects hydrologic basins, as is seen in the western populations of anodontine freshwater mussels (Mock et al. 2010). The host fishes of *Cyprogenia* that have been identified via laboratory studies include: *Cottus carolinae, Etheostoma blennioides*,* E. caeruleum*,* E. flabellare*,* E. spectabile squamosum*,* Percina caprodes*,* P. phoxocephala,* and *P. roanoka* (Jones and Neves 2002; Eckert 2003), and all of these fishes occupy habitats that are typical of the highland regions inhabited by *Cyprogenia*. The restricted movement of host fishes for *Cyprogenia* between the three highland regions is supported by population genetic analyses of a number of fish species that indicate reduced gene flow between and within these same basins (i.e. Echelle et al. 1975; Turner et al. 1996; Turner and Trexler 1998; Ray et al. 2006; Haponski et al. 2009). Limited movement of host fishes between basins would restrict gene flow between mussels inhabiting these same basins, and additional evidence for limited geneflow between mussel populations in different basins is seen in the reduced suitability of allopatric versus sympatric host‐fishes (Eckert 2003). In that study, fishes that were sympatric with the mussels tested transformed a higher proportion of mussel larvae to the juvenile stage than fishes of the same species collected in different basins. The lack of shared mtDNA haplotypes between *Cyprogenia* inhabiting the three regions and the high degree of differentiation at the microsatellite loci are consistent with biogeographic scenarios that these regions became isolated during the late Miocene or Pliocene (Hoagstrom et al. 2014).

### Species delimitation and conservation implications

Freshwater mussels are among the most endangered faunas in North America, and species delimitation is an important first step in understanding the significance of variation in conchological characteristics, reproductive strategies, habitats, and host fish requirements for these understudied organisms. Accurate delimitation of evolutionary lineages is important for the efficient use of conservation resources and the long‐term preservation of biodiversity. Within *Cyprogenia* the Eastern Highland group identified in our study conforms to the existing range of *C. stegaria*. *Cyprogenia stegaria* is a federally endangered species (USFWS 1990) and reproducing populations are now restricted to the Licking, Green, and Salt rivers in Kentucky and the Clinch River in Tennessee and Virginia. Within the former range of *C. aberti*, two distinct clusters occur. The Ouachita cluster includes populations from the Ouachita and Saline rivers, whereas the Ozark cluster includes populations from Black, St. Francis, and Spring (AR) rivers. Harris et al. (2009) recommended that the status of *C. aberti* in Arkansas be changed from Threatened to Endangered, and our results indicate that a further re‐examination of the conservation status of this species is warranted. At the present time, we recommend that Ozark and Ouachita clusters be treated as distinct evolutionarily significant units (ESUs) sensu Crandall et al. (2000) due to lack of ecological exchangeability as evidenced by apparent adaptation to local host fishes (Eckert 2003) and genetic differentiation demonstrated in this study. Such a designation would recognize the ecological and genetic distinctiveness of these entities for management purposes until additional morphological and genetic data can be used to more directly test their status as distinct species. The genetic data generated for each of the sampling sites indicates that at present, the levels of genetic diversity as measured by allelic richness and expected heterozygosity are high, and there is an absence of substantial inbreeding at all sites sampled. Any plans to propagate and translocate individual *Cyprogenia* should not include transfer of individuals between these three distinct lineages and potentially risk introducing new alleles that may result in hybridization and out‐breeding depression, which could have detrimental consequences.

## Conclusions

A substantial number of phylogeographic and population genetic studies have been conducted on freshwater mussels using mitochondrial markers (e.g. King et al. 1999; Roe et al. 2001; Roe 2013; Zanatta and Harris 2013). A standard assumption is that mitochondrial genes are largely neutral markers and as such are well‐suited to reconstructing the evolutionary relationships of organisms (Avise et al. 1987). Our research provides another example that mitochondrial markers are not always neutral with respect to natural selection, and so may reflect a biased evolutionary history. Mitochondrial and nuclear markers in *Cyprogenia* revealed two very different geographic patterns, and our investigation indicates that the DNA sequences of the mitochondrial ND1 gene are highly correlated with the color of the conglutinate lures in *Cyprogenia* and tests we conducted are consistent with balancing selection as the mechanism by which these mt DNA lineages were maintained over time. An investigation of the molecular basis for conglutinate colors and the impact of conglutinate colors on host‐fish choice have the potential to further test this hypothesis. Based on the analysis of 10 microsatellite loci, we conclude that there are currently three independent evolutionary entities in *Cyprogenia* and we recommend that these are treated as a distinct species in the case of *C. stegaria*, and ESUs in the case of the entities in the Ozark and Ouachita basins. We are currently studying conchological variation in *Cyprogenia* shells using 3D morphometrics to compare shell morphology within and between the lineages defined by our genetic analyses. We are also investigating the basis of conglutinate colors and the relationship between conglutinate color and mitochondrial variation in hopes of improving our understanding of this fascinating system.

## Conflict of Interest

None declared.

## Data Accessibility

DNA sequences: GenBank accessions KU687119–KU687320. Final DNA sequence assembly uploaded as online Supporting Information Sampling locations and microsatellite genotypes: Dryad.

## Supporting information


**Table S1.** Sample sizes from all *Cyprogenia* sampling locations included in this study. A total of 223 *Cyprogenia* individuals were included. DNA of 26 *C. aberti* and two *C. stegaria* (individuals from sites 1–14, and 18) were obtained from Serb (2006). DNA of 53 *C. stegaria* individuals (sites 15–18) were obtained from Grobler et al. (2011). The remaining 144 *C. aberti* individuals (sites 3–7, 12, 14) were collected by the authors.
**Table S2.** Outgroup taxa included in the Bayesian analysis with GenBank accession numbers.
