# Supplementary material for: Incongruence between mtDNA and nuclear data in the freshwater mussel genus Cyprogenia (Bivalvia: Unionidae) and its impact on species delineation
Source: Ecol Evol. 2016 Mar 11;6(8):2439–52. doi: 10.1002/ece3.2071 (PMC4788976; doi:10.1002/ece3.2071)
Supplement: Supplementary file 1 — Table S1. Sample sizes from all Cyprogenia sampling locations included in this study. A total of 223 Cyprogenia individuals were included. DNA of 26 C. aberti and two C. stegaria (individuals from sites 1–14, and 18) were obtained from Serb (2006). DNA of 53 C. stegaria individuals (sites 15–18) were obtained from Grobler et al. (2011). The remaining 144 C. aberti individuals (sites 3–7, 12, 14) were collected by the authors. Table S2. Outgroup taxa included in the Bayesian analysis with GenBank accession numbers. [file ECE3-6-2439-s001.docx]

**Table S1.** Sample sizes from all *Cyprogenia* sampling locations included in this study. A total of 223 *Cyprogenia* individuals were included in this study. DNA of 26 *C. aberti* and two *C. stegaria* (individuals from sites 1-14, and 18) were obtained from Serb (2006). DNA of 53 *C. stegaria* individuals (sites 15-18) were obtained from Grobler et al. (2011). The remaining 144 *C. aberti* individuals (sites 3-7, 12, 14) were collected by the authors.

| Species | Site ID | Drainage | State | Sample # | Source of samples |
| --- | --- | --- | --- | --- | --- |
| *C. aberti* | 1 | Fall River | KS | 2 | Serb (2006) |
|  | 2 | Spring River | KS | 1 | Serb (2006) |
|  | 3 | St. Francis River | MO | 27 | This study, Serb (2006) |
|  | 4 | Black River | MO | 28 | This study, Serb (2006) |
|  | 5 | Black River | AR | 17 | This study, Serb (2006) |
|  | 6 | Spring River | AR | 8 | This study, Serb (2006) |
|  | 7 | Spring River | AR | 17 | This study, Serb (2006) |
|  | 8 | Current River | AR | 1 | Serb (2006) |
|  | 9 | Buffalo River | AR | 1 | Serb (2006) |
|  | 10 | Strawberry River | AR | 2 | Serb (2006) |
|  | 11 | White River | AR | 1 | Serb (2006) |
|  | 12 | Ouachita River | AR | 23 | This study, Serb (2006) |
|  | 13 | Caddo River | AR | 5 | Serb (2006) |
|  | 14 | Saline River | AR | 13 | This study, Serb (2006) |
| *C. stegaria* | 15 | Licking River | KY | 23 | Grobler et al. (2011) |
|  | 16 | Salt River | KY | 8 | Grobler et al. (2011) |
|  | 17 | Green River | KY | 8 | Grobler et al. (2011) |
|  | 18 | Clinch River | TN | 8 | Grobler et al. (2011), Serb (2006) |

**Table S2.** Outgroup taxa included in the Bayesian analysis with GenBank accession numbers:

*Actinonaias ligamentina* (AY655085), *Amblema plicata* (AY158796), *Dromus dromas* (AY158750), *Epioblasma brevidens* (AY094378), *Epioblasma capsaeformis* (DQ208591), *Lampsilis cariosa* (EF446096), *Lampsilis fasciola* (DQ220721), *Lampsilis higginsii* (EF213061), *Lampsilis ornate* (AY158748), *Lampsilis ovata* (AY613797), *Lampsilis siliquoidea* (AY158747), *Lemiox rimosus* (AY655104), *Ligumia recta* (EF213055), *Medionidus conradicus* (AY158746), *Obliquaria reflexa* (AY158751), *Potamilus alatus* (AY655119), *Ptychobranchus fasciolaris* (AY655120), *Villosa iris* (DQ445185).
